# A Recombinant Oncolytic Pseudorabies Virus Expressing *Interleukin-18*, *Interferon-Gamma* and *PH20* Genes Promotes Systemic Antitumor Immunity

**DOI:** 10.3390/microorganisms11071850

**Published:** 2023-07-21

**Authors:** Xiaohui Han, Jingshuai Sun, Xiaocheng Lv, Xiaoyu Tang, Yubin Zheng, Jinyun Ma, Yuan Sun

**Affiliations:** 1 Guangdong Provincial Key Lab of Agro-Animal Genomics and Molecular Breeding, College of Animal Science, South China Agricultural University, Guangzhou 510642, China; hxh@stu.scau.edu.cn (X.H.); jssun@stu.scau.edu.cn (J.S.); lxc5860@stu.scau.edu.cn (X.L.); txy@stu.scau.edu.cn (X.T.); astyb@stu.scau.edu.cn (Y.Z.); 2 Guangdong Laboratory for Lingnan Modern Agriculture, Guangzhou 510642, China

**Keywords:** oncolytic virus, PRV, *IL-18*, *IFN-γ*, *PH20*

## Abstract

Pseudorabies virus (PRV) is considered to be a promising oncolytic virus that has potential as a cancer gene therapy drug. In this study, PRV-DCD-1-70 was used as a vector to carry exogenous genes *IL-18*, *IFN-γ* and *PH20* to construct novel recombinant PRV, rPRV-PH20 and rPRV-IL-18-γ-PH20, and their tumorolytic effects were evaluated in vitro and in vivo. Our study showed that recombinant PRV lysed all four tumor cell lines, Pan02, EMT-6, CT26 and H446, and rPRV-IL-18-γ-PH20 showed the best tumor lysis effect. Further studies in mice bearing Pan02 tumors showed that recombinant PRV, especially rPRV-IL-18-γ-PH20, were able to inhibit tumor growth. Moreover, an immunohistochemical analysis indicated that the recombinant PRV effectively increased the infiltration of CD4^+^T and CD8^+^T cells and enhanced the anti-tumor immune response of the organism in vivo. Overall, PRV carrying *PH20* and *IL-18-γ* exogenous genes demonstrated anti-tumor effects, providing a foundation for the further development and application of PRV as a novel tumor oncolytic virus vector.

## 1. Introduction

Oncolytic viruses (OVs) refer to a group of viruses that have either been genetically modified or occur naturally, with the ability to specifically target, infect, and destroy cancer cells while having minimal or no impact on healthy cells [1]. Viruses in the Alphaherpesvirus subfamily, such as herpes simplex virus 1 (HSV-1), herpes simplex virus 2 (HSV-2), and bovine herpesvirus-1 (BHV-1), have been extensively studied due to their potential to treat tumors [2,3,4,5]. In 2015, the US Food and Drug Administration (FDA) approved an HSV-1 developed by BioVex for the treatment of melanoma. In 2021, a genetically engineered herpes simplex virus 1 received conditional approval from Japan’s Ministry of Health, Labour and Welfare (MHLW) as oncolytic virotherapy for the treatment of patients with malignant glioma [6,7]. However, current oncolytic viruses still have drawbacks such as limited response rates, and new oncolytic viruses and new strategies need to be developed.

Porcine pseudorabies virus is a member of the Herpesviridae family and falls under the Alphaherpesvirinae subfamily [8]. PRV has not been widely explored for its ability to lyse cells and its role as a vector for cancer gene therapy. As a promising gene carrier for the treatment of cancer, PRV has various advantages. Firstly, PRV has a large genome and deletes virulence genes, including *TK*, *PK*, *gG*, *gD*, *gI* and *gE*, replacing them with exogenous genes that do not affect its replication and transmission [4,5,7]. In addition, PRV vaccine strains have been used for decades and have been demonstrated with a high level of safety and efficacy in animals [7]. The insertion of exogenous genes to arm oncolytic viruses is a common strategy to enhance tumor therapy. Hyaluronic acid (HA), also known as vitreous acid, is found in large quantities in the extracellular matrix (ECM) and is an important component of the tumor microenvironment (TME) [9]. HA can create a specific microenvironment conducive to tumor angiogenesis, invasion, and metastasis [10]. The presence of HA is closely linked to aggressive tumor behavior and an unfavorable prognosis in different types of tumors, including breast, colorectal, gastric, prostate, pancreatic and ovarian cancers [11]. The primary pathway for HA degradation is through enzymatic action by hyaluronidase (HAase). The most active human hyaluronidase is *PH20*, also known as sperm adhesion molecule 1 (*SPAM1*). The combination of *PH20* with CAR-T cells or oncolytic virus has been identified that could reduce tumor interstitial pressure and effectively promote drug diffusion, resulting in better anti-cancer effects [12,13].

*Interleukin 18* (*IL-18*) is a cytokine secreted by activated dendritic cells and intrinsic effector cells, which has been reported to be able to stimulate the immune system by inducing the production of *IFN-γ*, *GM-CSF*, *TNF-α* and *IL-18* in immunocompetent cells [14]. Additionally, it can enhance lymphocyte-mediated killing and increases the expression of particular chemokine receptors [15]. *Interferon-gamma* (*IFN-γ*) is a pro-inflammatory cytokine produced mainly by T cells and NK cells, among others, and plays an important role in regulating intrinsic and acquired immune responses in the tumor microenvironment [16]. The administration of therapeutically high doses of recombinant human *IL-18* significantly improves antitumor efficacy by stimulating cytotoxic lymphocytes to secrete more *IFN-γ*, enhancing particle-mediated cytotoxicity and increasing the expression of Fas ligands [17]. Therefore, *IL-18* and *IFN-γ* are promising candidates for the immunogene therapy of cancer.

The ability to carry large and multiple transgenes in the viral genome is one of the advantages of PRV vectors. Although PRV has shown great potential for oncology therapy, previous studies have not manipulated the virus through genetic engineering to express specific genes. We aimed to study whether PRV expressing *IL-18*, *IFN-γ* and *PH20* could infect and kill cancer cell lines and promote antitumor immunity. In this study, we used Red/ET recombinant technology combined with a site-specific recombination system to construct recombinant PRV and performed in-depth in vivo and in vitro evaluations of their oncolytic effects, which would provide useful information to establish a scientific foundation for the future advancement and utilization of PRV as a virus vector for oncolytic purposes.

## 2. Materials and Methods

### 2.1. E. coli Strains and Plasmids

Engineering bacteria such as GBdir-Pir116, *E. coli* GB08-red, pSC101-BAD-Cre-tet in *E. coli* GB2005 and R6K-TK-HA-hCMV-Amp-ccdB-pA-lox66-kan-ter-lox71-BGH plasmid were presented by the Shandong University-Helmholtz Institute of Biotechnology, State Key Laboratory of Microbial Technology.

pBeloBAC11-DCD1-70-ΔgG-IL-18-γ-ΔgEgI-FRT and pBeloBAC11-DCD1-70#-ΔgEgI-FRT plasmids were maintained by our laboratory.

*PH20* in pUC-GW-Amp, a plasmid carrying the *PH20* gene, was synthesized by Azenta Life Sciences (Suzhou, China).

### 2.2. Cells and Viruses

PRV-DCD-1-70 (GenBank accession no. OL639029), pBeloBAC11-DCD1-70-ΔgG-IL-18-γ-ΔgEgI-FRT (rPRV-IL-18-γ) and pBeloBAC11-DCD1-70#-ΔgEgI-FRT and pBeloBAC11-amp-DCD1-70#-ΔgG-rox2332-ΔTK-loxP-ΔgEgI-FRT (rPRV-gG^−^-TK^−^-gEgI^−^) used in this study were all maintained by our laboratory [18].

The cell lines used in the experiment, including Vero, PK-15, Pan02, EMT-6, CT26 and H446, were preserved in the Key Laboratory of Animal Health Aquaculture and Environmental Control, South China Agricultural University and cultured in a constant-temperature incubator at 37 °C with 5% CO_2_ for passaging.

### 2.3. Construction of PRV Recombinant Virus

The template used for the PCR amplification of the *PH20* gene was the *PH20* in the pUC-GW-Amp plasmid. The purpose of the amplification was to attach the homologous arms of the intermediate transfer vector R6K-TK-HA-hCMV-Amp-ccdB-pA-lox66-kan-ter-lox71-BGH to the left and right sides of *PH20*. Table 1 contained the PCR amplification primers for the exogenous genes that carry the homologous arm of the *TK* locus. The resulting PCR products were analyzed by agarose gel electrophoresis, and the gel was saved for future use.

To obtain recombinant bacteria containing R6K-TK-HA-hCMV-PH20-lox66-kan-ter-lox71-BGH, the intermediate transfer vector R6K-TK-HA-hCMV-Amp-ccdB-pA-lox66-kan-ter-lox71-BGH was digested with NheI to produce a linearized plasmid fragment. This fragment was then subjected to linear homologous recombination (LLHR) with the PH20 gel recovery product in GBdir-Pir116-engineered bacteria.

To obtain a linearized fragment containing homologous arms on both sides of the PRV *TK* locus, the recombinant plasmid R6K-TK-HA-hCMV-PH20-lox66-kan-ter-lox71-BGH was cleaved with AscI. This fragment, named TK-HA-hCMV-PH20-lox66-kan-ter-lox71, was then subjected to linear loop homologous recombination with our laboratory-conserved plasmids, namely pBeloBAC11-DCD1-70#-ΔgEgI-FRT and pBeloBAC11-DCD1-70#-ΔgG-IL-18-γ-ΔgEgI-FRT, respectively. These reactions were carried out in *E. coli* GB08-red receptor state, followed by plasmid extraction and electrotransferral into pSC101-BAD-Dre-tet in *E. coli* GB2005. The recombinant plasmids pBeloBAC11-DCD1-70#-ΔTK-PH20-ΔgEgI-FRT (rPRV-PH20) and pBeloBAC11-DCD1-70#-ΔgG-IL-18-γ-ΔTK-PH20-ΔgEgI-FRT (rPRV-IL-18-γ-PH20) were obtained by inducing the Cre enzyme to eliminate Kan resistance. These plasmids carry the gene for *IL-18-γ* and *PH20*. More detailed information for constructing plasmids was described in a previous study [18].

### 2.4. Rescue and Validation of Recombinant Viruses

To rescue the attenuated mutants rPRV-PH20 and rPRV-IL-18-γ-PH20, the PRV recombinant viruses were transfected into Vero cells using Lipofectamine™ 3000. The recombinant virus was then verified via PCR using primers that flank either the *gG* or *TK* sites (Table 2).

### 2.5. Cell Viability Assay

Tumor cells in the logarithmic phase were selected and inoculated into 96-well plates at a density of 5^10^–10^10^ cells per well. After 24 h of inoculation, each group of PRV recombinant viruses was diluted to different MOIs (0.01, 0.1, 1, 10) with DMEM containing 2% FBS and inoculated onto culture plates. The control group was treated with serum-only and virus-free medium and cultured in a constant-temperature incubator. After 48 h of inoculation, 10% CCK 8 reagent (Abmole Bioscience, Houston, TX, USA) was added to the medium and incubated in a dark incubator for 1–2 h. The absorbance at 450 nm was measured using an enzyme-labeled instrument (Thermo Scientific Multiskan™ FC, Waltham, MA, USA).

### 2.6. Animal Experiments

In this study, 6-week-old SPF-grade C57BL/6 female mice were purchased from Zhuhai BesTest Bio-Tech Co., Ltd. (Zhuhai, China), with the animal quality license number of SYXK (Guangdong) 2022-0136. After 5 days of adaptive feeding in the Experimental Animal Center of South China Agricultural University, the experiment was carried out. Each mouse’s right axilla was injected with 1 × 10^6^ cells/100 μLPan02 cell suspension. When the tumor volume grew to 50–100 mm^3^, the mice were divided into five groups. There was no statistical difference in tumor volume between the groups before treatment. Intratumoral injections of 1 × 10^7^ TCID_50_/mL of rPRV-gG^−^-TK^−^-gEgI^−^, rPRV-IL-18-γ, rPRV-PH20 or rPRV-IL-18-γ-PH20 were administered, with PBS treatment serving as the control (100 µL). Treatment was carried out 5 times with a 3-day interval between each treatment. The tumor volume was measured and recorded every three days using an electronic vernier caliper to generate a tumor growth curve.

### 2.7. Histopathological Examination of Tumor

The tumor tissue samples were fixed in a 4% paraformaldehyde solution, followed by embedding in paraffin and sectioning. The paraffin sections were deparaffinized and subjected to staining with hematoxylin and eosin solution. The sections were then gradually dehydrated with absolute ethanol, n-butanol and xylene and finally sealed with neutral gum for microscopic examination.

### 2.8. Immunohistochemical Analysis of Tumor

The tumor tissue samples were fixed in 4% paraformaldehyde, embedded in paraffin and sectioned. The tissue sections were then dewaxed and subjected to antigen retrieval by placing them in citric acid antigen repair solution and heating them in a microwave oven for 10 min. Endogenous peroxidase was blocked by immersing the sections in 3% hydrogen peroxide solution for 10 min, followed by washing with PBS and spin-drying. The sections were blocked with 3% BSA and then incubated with primary antibody overnight at 4 °C. After that, secondary antibodies corresponding to the species of the primary antibody were incubated for 1 h. The sections were then stained with DAB-developing solution and hematoxylin to visualize the nuclei. Finally, the sections were dehydrated and observed under a microscope. The positive expression of DAB appeared brownish yellow, while the nuclei stained with hematoxylin appeared blue. Image Pro Plus 6.0 (Media Cybernetics, Rockville, MD, USA) was used to calculate the integrated optical density (IOD) of the IHC section.

### 2.9. Statistical Analysis

Data are expressed as mean ± standard error (SEM). Two-way repeated measures ANOVA was performed on mouse tumor volumes using GraphPad Prism 7.0 software, and CCK8 data were analyzed using one-way ANOVA with significance expressed as *p* values, with * *p* < 0.05 representing significant differences and ** *p* < 0.01 and *** *p* < 0.001 representing highly significant differences.

## 3. Result

### 3.1. Construction of PRV Recombinant Virus Infectious Clones and Virus Rescue

**Figure 1:**
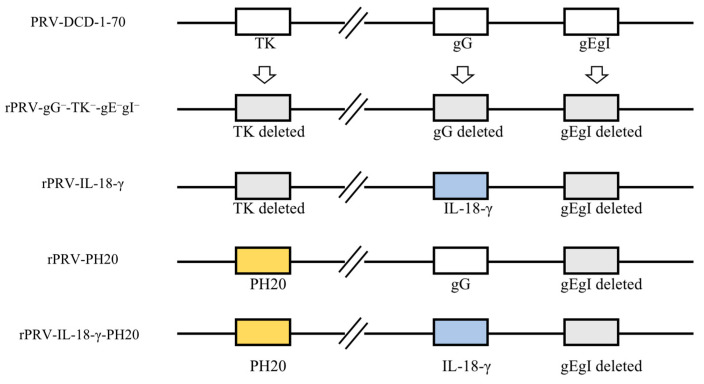
The recombinant PRV vectors were constructed based on the PRV−DCD−1−70 strain. Modifications include *TK*, *gG*, *gE* and *gI* gene deletion and the insertion of the related transgenes.

The recombinant viruses rPRV-PH20 and rPRV-IL-18-γ-PH20 were constructed from PRV-DCD-1-70. The *TK* gene was replaced and modified with the *PH20* gene based on the original plasmids of pBeloBAC11-DCD1-70#-ΔgEgI-FRT and pBeloBAC11-DCD1-70#-ΔgG-IL-18-γ-ΔgEgI-FRT. The final recombinant plasmids pBeloBAC11-DCD1-70#-ΔTK-PH20-ΔgEgI-FRT (rPRV-PH20) and pBeloBAC11-DCD1-70#-ΔgG-IL-18-γ-ΔTK-PH20-ΔgEgI-FRT (rPRV-IL-18-γ-PH20) were obtained. Recombinant viruses rPRV-gG^−^-TK^−^-gEgI^−^ and rPRV-IL-18-γ were originally constructed and preserved for our laboratory [18]. The modification schemes of the four recombinant viruses rPRV-gG^−^-TK^−^-gEgI^−^, rPRV-IL-18-γ, rPRV-PH20 and rPRV-IL-18-γ-PH20 are shown in Figure 1.

**Figure 2:**
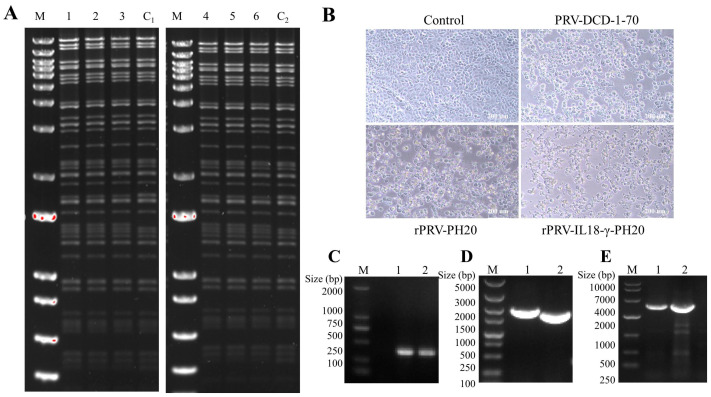
Validation and characterization of the infectious clones. (**A**) PvuII and NheI restriction enzyme analysis of the rPRV-PH20 and rPRV-IL-18-γ-PH20. M, 1kb DNA ladder (Thermo Fisher, Waltham, MA, USA); lanes 1–3, pBeloBAC11-cm-amp-PRV-DCD1-ΔgEgI-FRT-ΔTK-PH20-lox66-Δkan; lane C1, pBeloBAC11-cm-amp-PRV-DCD1-ΔgEgI-FRT-ΔTK-PH20-lox66-kan; lanes 4–6, pBeloBAC11-cm-amp-PRV-DCD1-ΔgEgI-FRT-ΔgG-IL-18-γ-ΔTK-PH20-lox66-Δkan; lane C2, pBeloBAC11-cm-amp-PRV-DCD1-ΔgEgI-FRT-ΔgG-IL-18-γ-ΔTK-PH20-lox66-kan. (**B**) Typical cytopathic effects (CPE) of PRV-DCD-1-70, rPRV-PH20 and rPRV-IL-18-γ-PH20 infection in Vero. (**C**–**E**) represent recombinant viruses validated with primers for the *gB*, *gG* and *TK* sites; lan 1, rPRV-PH20; lane 2, rPRV-IL-18-γ-PH20.

The recombinant plasmids rPRV-PH20 and rPRV-IL-18-γ-PH20 were further identified using PvuII and NheI digestion, and the results showed that the enzymatic cleavage of that recombinant plasmid was consistent with the mimic enzymatic cleavage, which verified that the recombinant viruses rPRV-PH20 and rPRV-IL-18-γ-PH20 were constructed correctly (Figure 2A). Then, Vero cells were transfected with the recombinant viruses for 48 or 72 h, and the results showed that these cells displayed evident lesions with the rounding up and clustering of cells, as well as cell shedding and floating after death, which were analogous with those of the wild virus PRV-DCD1-70 (Figure 2B). The recombinant plasmids were further identified by PCR and amplified with verification primers corresponding to the *gB*, *gG* and *TK* sites to detect their stabilities. As shown in Figure 2C, both recombinant plasmids rPRV-PH20 and rPRV-IL-18-γ-PH20 were able to successfully amplify the *gB* fragment, the conserved region of PRV, with the expected length. The *gG* gene of recombinant plasmids rPRV-PH20 was not modified by substitution, and its original length was 2151 bp, but in the recombinant plasmid rPRV-IL-18-γ-PH20, the length of *gG* replaced with the *IL-18-γ* changed to 2010 bp (Figure 2D). The *TK* sites of both plasmids were modified with the *PH20* gene, and the length of the bands became 3501 bp (Figure 2E). After the PCR product was electrophoresed on agarose gel, the band size was as expected, and the plasmid was sent to Shanghai Sangon Biotech (Shanghai, China) for correct sequencing, and the results indicated that the two recombinant plasmids rPRV-PH20 and rPRV-IL-18-γ-PH20 were constructed successfully.

### 3.2. Pathological Characteristics of PRV Recombinant Virus on Various Tumor Cell Lines

**Figure 3:**
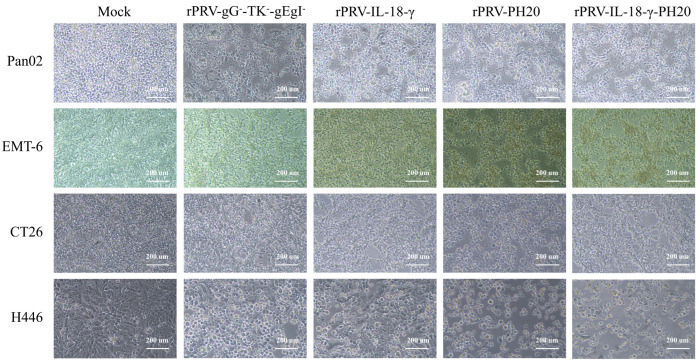
Image characteristics of recombinant PRV after the infection of various tumor cells. Pan02, EMT-6, CT26 and H446 cells were infected with rPRV-gG^−^-TK^−^-gEgI^−^, rPRV-IL-18-γ, rPRV-PH20 and rPRV-IL-18-γ-PH20 at an MOI of 1. The images were obtained 48 h after infection.

To initially assess whether the recombinant PRV have an oncolytic effect on certain tumor cell lines, the pathological features of the recombinant viruses in each tumor cell line were observed using light microscopy. After infecting mouse pancreatic cancer Pan02, mouse breast cancer EMT-6, mouse colon cancer CT26 and human small-cell lung cancer H446 tumor cells with separate sets of PRV recombinant viruses at a multiplicity of infection (MOI) of 1, the resulting outcomes were obtained (Figure 3). Following a 48 h incubation period, untreated cells in the control group exhibited strong adhesion and close arrangement. In contrast, tumor cells infected with rPRV-gG^−^-TK^−^-gEgI^−^, rPRV-IL-18-γ, rPRV-PH20 and rPRV-IL-18-γ-PH20 displayed varying degrees of lysis, manifested by morphological features such as rounding and rupturing. Additionally, the infected cells exhibited a reduced cell area, loose intercellular substance and significant changes in morphology. These results indicated that the above four recombinant PRV were effective in killing Pan02, EMT-6, CT26 and H446 cells.

### 3.3. Oncolytic Spectrum of Recombinant PRV In Vitro

**Figure 4:**
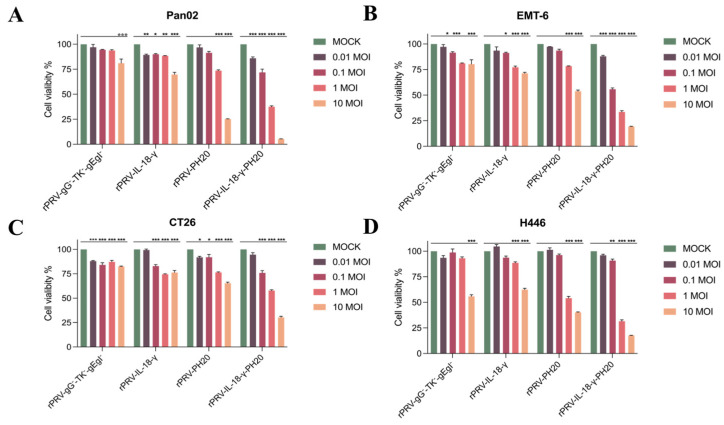
PRV recombinant viruses induced tumor cell death in vitro (**A**–**D**). Pan02 (**A**), EMT-6 (**B**), CT26 (**C**) and H446 (**D**) were infected with PRV recombinant viruses at the indicated MOI for 48 h. The viability of different cells was measured via CCK-8 assay. * *p* < 0.05; ** *p* < 0.01; *** *p* < 0.001. Data are shown as mean ± SEM.

In order to thoroughly evaluate the cytotoxic effects of different PRV recombinant viruses on various tumor cell lines, four separate sets of recombinant viruses were used. Each set was applied to infect the Pan02, EMT-6, CT26 and H446 tumor cells using different MOI levels of 0.01, 0.1, 1 and 10, respectively. After 48 h, the CCK8 assay was used to determine the tumor cell viability. As shown in Figure 4, all PRV recombinant viruses effectively reduced the cell activities of the Pan02, EMT-6, CT26 and H446 tumor cell lines, exhibiting a high potential for in vivo tumor eradication. After 48 h of infection with the recombinant viruses rPRV-gG^−^-TK^−^-gEgI^−^, rPRV-IL-18-γ, rPRV-PH20 and rPRV-IL-18-γ-PH20 with an MOI = 10, the mean cell survival rates of the Pan02 cell lines were 81.04%, 69.68%, 25.26% and 5.53% (Figure 4A); the mean cell viability of the EMT-6 cell line was 80.34%, 71.31%, 53.89% and 19.31% (Figure 4B); the mean cell viability of the CT-26 cell line was 82.53%, 76.31%, 65.37% and 30.37% (Figure 4C) and the mean cell survival rates for the H446 cell line were 55.87%, 62.45%, 40.25% and 17.50%, respectively (Figure 4D). The killing effect of recombinant PRV on each tumor cell line was significantly enhanced after the loading of exogenous gene *IL-18-γ* or *PH20*. Among them, the recombinant PRV rPRV-IL-18-γ-PH20 loaded with both *IL-18-γ* and *PH20* showed the best oncolytic effect. After infecting the above four tumor cell lines with 10 MOI of rPRV-IL-18-γ-PH20 for 48 h, the survival rate of each tumor cell was less than 30%. Due to its increasing sensitivity to all PRV recombinant viruses at an MOI of 10, the Pan02 cell line was chosen for further studies in vivo.

### 3.4. Antitumor Effect in Pan02 Tumor Models

**Figure 5:**
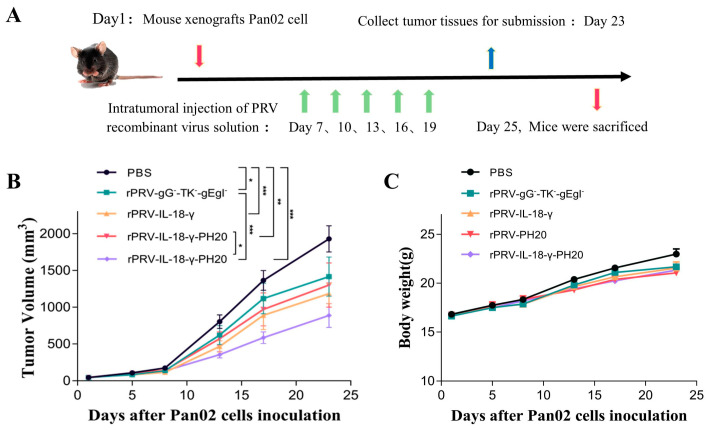
Oncolytic efficacy of PRV recombinant virus in C57BL/6 mice. (**A**) Therapeutic scheme. An amount of 100 µL of PBS or PRV recombinant virus (1 × 10^7^ TCID50/mL) was injected into the established tumor on days 7, 10, 13, 16 and 19. (**B**) Mean tumor volumes in mice treated with PBS, rPRV-gG^−^-TK^−^-gEgI^−^, rPRV-IL-18-γ, rPRV-PH20 or rPRV-IL-18-γ-PH20 (* *p* < 0.05, ** *p* < 0.01, *** *p* < 0.001). (**C**) Mean body weights of mice in each group. Data were shown as mean ± SEM.

To further evaluate the anti-tumor effect of PRV recombinant viruses in vivo, the Pan02-bearing mouse model was constructed, and mice were randomly assigned to five groups with each group containing 10 mice. The Pan02-bearing mice in five groups were administered with identical doses of rPRV-gG^−^-TK^−^-gEgI^−^, rPRV-IL-18-γ, rPRV-PH20, rPRV-IL-18-γ-PH20 and PBS, respectively. The treatment was administered every 3 days starting from the 7th day of tumor loading, for a total of five times. Each oncolytic virus experimental group was treated via an intratumoral injection at a dose of 10^7^ TCID_50_/per needle (Figure 5A).

During the treatment period, the volume of tumors in the Pan02-bearing mice was monitored. By the 23rd day after tumor induction, the average tumor volume in the PBS group had reached 1929.68 mm^3^, which was almost 2000 mm^3^, leading to the termination of the study. The average tumor volumes in the mice treated with rPRV-gG^−^-TK^−^-gEgI^−^, rPRV-IL-18-γ, rPRV-PH20 and rPRV-IL-18-γ-PH20 were 1415.10 mm^3^, 1183.94 mm^3^, 1226.56 mm^3^ and 885.66 mm^3^, respectively. These findings suggested that all groups of PRV recombinant viruses exhibit a certain degree of tumor growth inhibition in the mouse Pan02 tumor model. Among them, the tumor volume of mice treated with recombinant virus rPRV-gG^−^-TK^−^-gEgI^−^ was reduced by one-quarter compared to the control group, and the tumor growth rate of mice treated with rPRV-IL-18-γ and rPRV-PH20 was further inhibited but did not reach a significant difference compared to rPRV-gG^−^-TK^−^-gEgI^−^. The PRV recombinant virus carrying both the IL-18-γ and PH20 exogenous genes displayed the most potent inhibition effect, with the tumor volume in this group of mice being less than half that of the control group by the time the measurement endpoint was reached. (Figure 5B). At the same time, the mice showed a slow and steady increase in body weight over time. Although there was no statistical difference in the mean body weight between groups (*p* > 0.05), the increasing body weight of the mice indicated that the recombinant PRV did not have a negative impact on the normal growth of mice in each group (Figure 5C).

### 3.5. Histopathological Examination of Tumor

**Figure 6:**
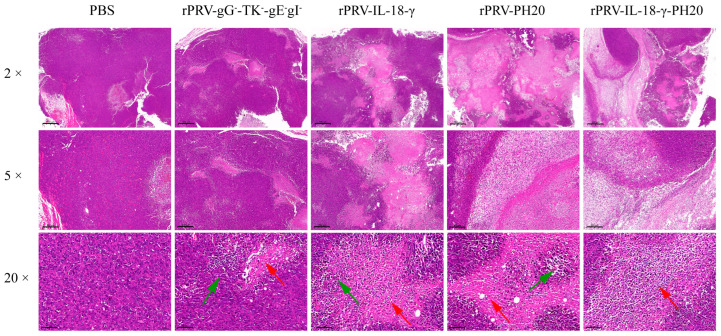
Histopathological findings of tumors in mice treated with PRV recombinant virus. The image displayed represents one mouse in each group, with 2×, 5× and 20× magnification. The red arrows in the figure indicate focal necrosis of tumor cells, and the green arrows indicate infiltration of tumor tissue by inflammatory cells.

On the 23rd day of the experiment, the tumors in the Pan02-bearing mice were collected and subjected to HE staining to assess the differences in the pathological characteristics among the tumor tissues. The results showed that the tumor cells treated with PRV recombinant viruses displayed obvious focal necrosis, especially cells infected with rPRV-IL-18-γ-PH20. In the rPRV-gG^−^-TK^−^-gEgl^−^-treated group, the overall structure of the tumor tissue was slightly abnormal. In the rPRV-IL18-γ-treated group and the rPRV-PH20-treated group, the areas of focal necrosis of tumor cells were increased. In the rPRV-IL18-γ-PH20-treated group, the overall structure of the tumor tissue was abnormal, and significant necrosis of large tumor cells was observed in the tissue, with only a part of the nuclear outline at the site of necrosis. No obvious tumor cell necrosis and lymphocyte infiltration were observed in the PBS-treated group, and no vascular congestion and dilation were observed in the tissue interstitium (Figure 6).

### 3.6. Immunohistochemical Analysis of Tumor

**Figure 7:**
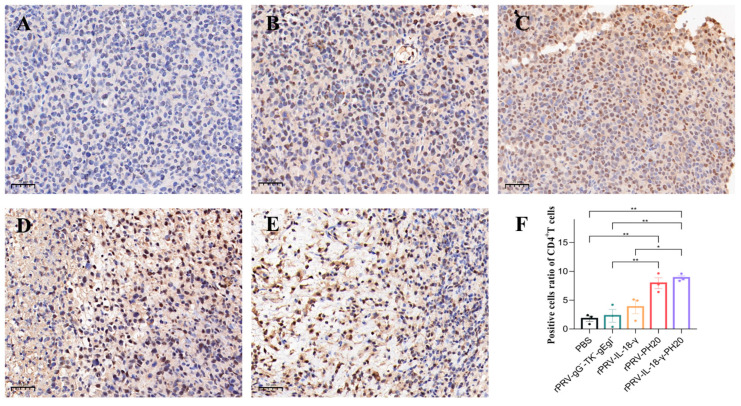
Immunohistochemical staining results of CD4^+^T cells in mouse tumor. The image displayed represents one mouse in each group, with 20× magnification. (**A**) PBS group. (**B**) rPRV-gG^−^-TK^−^-gEgI^−^-treated group. (**C**) rPRV-IL-18-γ-treated group. (**D**) rPRV-PH20-treated group. (**E**) rPRV-IL-18-γ-PH20-treated group. (**F**) Positive cell ratio of CD4^+^T cells. * *p* < 0.05; ** *p* < 0.01. Data are shown as mean ± SEM.

**Figure 8:**
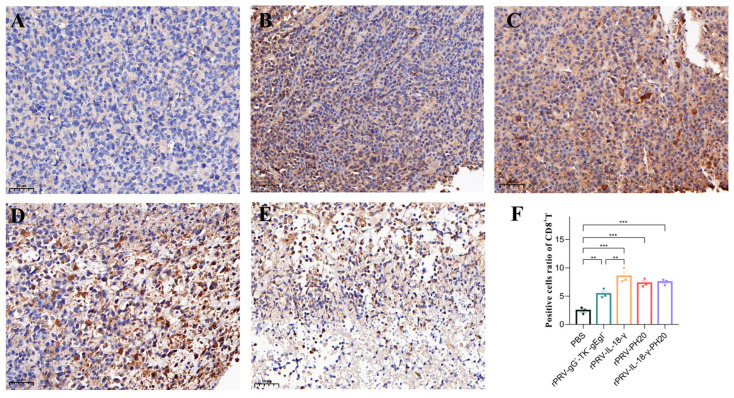
Immunohistochemical staining results of CD8^+^T cells in mouse tumor. The image displayed represents one mouse in each group, with 20× magnification. (**A**) PBS group. (**B**) rPRV-gG^−^-TK^−^-gEgI^−^-treated group. (**C**) rPRV-IL-18-γ-treated group. (**D**) rPRV-PH20-treated group. (**E**) rPRV-IL-18-γ-PH20-treated group. (**F**) Positive cell ratio of CD8^+^T cells. ** *p* < 0.01; *** *p* < 0.001. Data are shown as mean ± SEM.

To gain further insight into the impact of recombinant oncolytic PRV application on the tumor microenvironment, tumor tissues were harvested on the 23rd day and subjected to immunohistochemical staining for CD4^+^T cells, CD8^+^T cells and hyaluronidase. CD4^+^T cells participate in the anti-tumor immune response by secreting various cytokines and chemokines to recruit inflammatory cells and help activate CD8^+^T cells, while CD8^+^T cells mainly exert immunocidal effects through the perforin–granzyme pathway and the Fas-FasL pathway. Increasing the proportion of CD8^+^T cells helps suppress tumor development. The results showed that the cells in the PBS group exhibited a compact arrangement, complete morphology and light brown color. However, in the treatment groups of rPRV-gG^−^-TK^−^-gEgI^−^ rPRV-IL-18-γ, rPRV-PH20 and rPRV-IL-18-γ-PH20, the cells displayed diverse degrees of necrosis, a lighter color of nuclei, and a wider range of antigen staining. Compared to the PBS group, cells infected by the PRV recombinant viruses exhibited significantly increased infiltration of CD4^+^T and CD8^+^T cells, as indicated by deeper antigen staining (Figure 7 and Figure 8). These results indicated that the recombinant PRV in this study could effectively increase the infiltration of CD4^+^T and CD8^+^T cells in the Pan02-bearing mice to trigger an anti-tumor immune response in the body.

**Figure 9:**
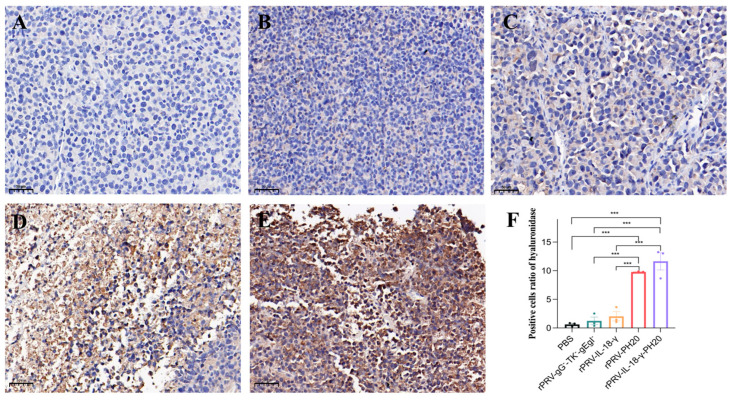
Immunohistochemical staining results of hyaluronidase in mouse tumor. The image displayed represents one mouse in each group, with 20× magnification. (**A**) PBS group. (**B**) rPRV-gG^−^-TK^−^-gEgI^−^-treated group. (**C**) rPRV-IL-18-γ-treated group. (**D**) rPRV-PH20-treated group. (**E**) rPRV-IL-18-γ-PH20-treated group. (**F**) Positive cell ratio of hyaluronidase. *** *p* < 0.001. Data are shown as mean ± SEM.

In tumor sections from the PBS group, the rPRV-gG^−^-TK^−^-gEgI^−^-treated group and the rPRV-IL-18-γ-treated group, the nuclei of cells were stained dark blue and appeared extremely light brown, indicating a lack of exogenous hyaluronidase expression. In contrast, cells infected by the rPRV-PH20 and rPRV-IL-18-γ-PH20 recombinant viruses showed shallower nuclear staining and extremely deep antigen staining, suggesting the successful expression of hyaluronidase by the recombinant PRV carrying the *PH20* gene (Figure 9).

## 4. Discussion

Presently, research regarding oncolytic viruses employing PRV as a vector is relatively scarce, with studies primarily conducting in vitro and in vivo tests using a virulence-gene-knockout PRV [5,7,18]. In this study, we used PRV as an oncolytic virus vector and carried out genetic engineering to construct PRV recombinant viruses carrying anti-tumor genes for the first time and verified their therapeutic effect on the mouse pancreatic cancer tumor model. The purpose of this study is to enhance both viral therapy and tumor immunotherapy by promoting viral spread and immune cell infiltration within the tumor while maintaining the oncolytic effect of the PRV. The results from studies in vitro and in vivo demonstrated that PRV-mediated *IL-18-γ* and *PH20* co-expression performed cooperative and enhanced antitumor effects. The results of the study will provide a scientific basis for the further development and application of PRV as an oncolytic virus.

Previous research has demonstrated that PRV-IE180 has the capacity to eliminate various types of tumor cells in vitro [10]. In addition, PRV with deleted *TK*, *gE* and *gI* virulence genes has been reported to possess the ability to impede tumor growth and prolong the survival time of mice in bladder cancer and colon cancer models, while simultaneously considering the efficacy and safety of oncolysis [5,19]. Our study also showed that the PRV with the *TK*, *gG*, *gE* and *gI* virulence genes deleted was effective in killing four tumor cell lines, including Pan02, EMT-6, CT26 and H446, thereby expanding the oncolytic spectrum of PRV in vitro. By administering intratumoral injections of the recombinant virus rPRV-gG^−^-TK^−^-gEgI^−^ to pancreatic-cancer-bearing mice, we observed that the virus significantly delayed the rapid growth of pancreatic cancer tumors. These findings indicated that PRV-attenuated viral strains possess significant anti-tumor potential as a recombinant tumor oncolytic vector. However, the safety of oncolytic viruses is of greater concern to many researchers, and it is of concern that PRV can infect both human cancer cells and epithelial cells [5], which may pose a risk if used in human tumor therapy. In future studies, we will carry out animal toxicity experiments to evaluate the safety of PRV recombinant virus and continue to optimize the treatment dosage and treatment time interval of oncolytic virus, so as to achieve the maximum treatment effect with minimum toxic and side effects.

Studies have shown that loading extracellular matrix (ECM)-degrading enzymes such as relaxin, metalloproteinase and hyaluronidase on oncolytic viruses can facilitate the penetration and dissemination of viruses and drugs within tumors [20,21,22,23]. Therefore, we selected hyaluronidase *PH20* as the first exogenous gene loaded on the PRV vector to reformulate the tumor microenvironment. In the present study, the results of HE staining and IHC staining obviously showed that rPRV-PH20 and rPRV-IL-18-γ-PH20, which carried the *PH20* gene, effectively induced widespread tumor cell necrosis, which was consistent with the study in which *PH20* is armed in oncolytic adenoviruses for treating glioblastoma and pancreatic cancer [12,23]. By breaking down hyaluronic acid, *PH20* can enhance the transport and permeation of molecules up to 200 nm in diameter [16]. This range of enhanced penetration may enable better distribution of the PRV throughout the tumor tissue, leading to increased viral potency and improved transmission within the tumor. Other studies have shown that the combination of hyaluronidase and oncolytic virus therapy not only enhances the penetration of the virus into the tumor but also reduces the tumor interstitial pressure and improves local blood perfusion [13,24]. Undoubtedly, the combination therapy of *PH20* and PRV deserves more studies to develop more effective oncolytic viruses.

The other two exogenous genes we chose were *IL-18* and *IFN-γ*. *IL-18* and *IFN-γ* are both pro-inflammatory cytokines, and both can exert anti-tumor effects by promoting the production of anti-cancer cytokines and enhancing the killing of tumors by immune cells [15,25,26]. *IL-18* has shown antitumor activity in preclinical animal models such as lung cancer, breast cancer, sarcoma and melanoma [17]. A variety of anti-tumor effects of *IFN-γ* have been confirmed, including regulating antigen presentation, promoting inflammatory signals, inhibiting tumor cell proliferation and anti-tumor angiogenesis [27]. Based on the above research. We replaced the *gG* gene of the PRV with the *IL-18-γ* gene, and the results of the cellular experiments showed that the recombinant virus rPRV-IL18-γ significantly reduced the survival of the Pan02, EMT-6, CT26 and H446 tumor cells compared with recombinant viruses without exogenous gene insertion. After treatment with rPRV-IL-18-γ, the growth rate of pancreatic cancer tumors in tumor-bearing mice was significantly reduced. Histopathological and immunohistochemical analyses showed that the recombinant virus rPRV-IL-18-γ increased the infiltration ratio of CD4^+^T cells, CD8^+^T cells and inflammatory cells in the tumors of pancreatic cancer mice. Accordingly, we speculated that the therapeutic effect of rPRV-IL-18-γ on pancreatic cancer in mice might be mediated by enhancing the anti-tumor immunity of the body. *IL-18* has been reported to synergistically induce *IFN-γ* production by CD8^+^ T and NK cells with *IL-12*, followed by a direct tumor-killing function through the Fas-FasL and TRAIL mechanisms [28,29]. In turn, *IFN-γ* can stimulate *IL-18* production by dendritic cells and accelerate the proliferation of memory CD8^+^T cells, thus creating a positive feedback loop [30]. Consequently, the linkage of the *IL-18* and *IFN-γ* genes could make them mutually reinforcing and may produce superimposed antitumor effects, but the mechanism of synergistic effects of *IL-18* and *IFN-γ* in antitumor needs to be elucidated by further studies.

Numerous studies have demonstrated the efficacy of *IL-18*, *IFN-γ* and *PH20* in cancer treatment [10,22,30,31,32,33,34], but the therapeutic potential of their combination has not been previously reported. Our hypothesis was that the co-administration of *IL-18*, *IFN-γ* and *PH20* with the PRV oncolytic virus would result in a superior antitumor effect based on the properties of these immunostimulatory molecules. Evidence of studies from in vitro and in vivo supported this hypothesis, indicating that the PRV-mediated co-expression of *IL-18-γ* and *PH20* produces a synergistic anti-tumor effect. *PH20* could enhance tissue permeability and improve the bioavailability of the PRV by degrading hyaluronic acid, reducing the restriction of the PRV by the tumor microenvironment when it spreads within the tumor. Meanwhile, *IL-18-γ* can recruit immune cells into the tumor microenvironment and activate and promote immune cell function, thereby reversing the immunosuppressive tumor microenvironment. Collectively, the simultaneous expression of *IL-18-γ* and *PH20* can effectively regulate the tumor tissue microenvironment and enable the PRV oncolytic virus to function optimally. Our research shows that PRV, as an oncolytic virus vector carrying genes to improve tumor microenvironment and immunostimulating genes, is a potential treatment for pancreatic cancer.

## Figures and Tables

**Table 1:** The primers of PCR amplification.

Primers	Sequences (5′ to 3′)
PH20-homologous arm-F	AGCTCTTAAGGCTAGAGTACTTAATACGACTCACTATAGGGCCACCATGGCTATGGGAGTGCTAAAATTCAAGCA
PH20-homologous arm-R	CGTCCCTATACTTTATTCATCATTTTAATGCTAAACGCCTTCAGAAGAAACCAATTCTGCT

**Table 2:** The primers of PCR validation.

Primers	Sequences (5′ to 3′)
gB-F	tcgacgatgcagttgacggag
gB-R	gtgctcttcaaggagaacatcg
gG-check-F	gtacgccgggacccatcgccag
gG-check-R	gttcagcagccggtccacctg
TK-check-F	ttgacttcaaaggccagggtcaag
TK-check-R	ggatgcgcgtgtcgttgag

## Data Availability

The data presented in this study are available upon request from the corresponding author.
